# Crystallographic Fragment Screening of a Bifunctional Proline Catabolic Enzyme Reveals New Inhibitor Templates for Proline Dehydrogenase and L-Glutamate-γ-semialdehyde Dehydrogenase

**DOI:** 10.3390/molecules29225408

**Published:** 2024-11-16

**Authors:** Kaylen R. Meeks, Alexandra N. Bogner, Jay C. Nix, John J. Tanner

**Affiliations:** 1 Department of Biochemistry, University of Missouri, Columbia, MO 65211, USA; kmcbp@missouri.edu (K.R.M.); bogneral@gmail.com (A.N.B.); 2 Molecular Biology Consortium, Advanced Light Source, Lawrence Berkeley National Laboratory, Berkeley, CA 94720, USA; jcnix@lbl.gov; 3 Department of Chemistry, University of Missouri, Columbia, MO 65211, USA

**Keywords:** X-ray crystallography, fragment-based lead discovery, enzyme inhibition, flavoenzyme, aldehyde dehydrogenase, proline catabolism

## Abstract

The proline catabolic pathway consisting of proline dehydrogenase (PRODH) and L-glutamate-γ-semialdehyde (GSAL) dehydrogenase (GSALDH) catalyzes the four-electron oxidation of L-proline to L-glutamate. Chemical probes to these enzymes are of interest for their role in cancer and inherited metabolic disease. Here, we report the results of a crystallographic fragment-screening campaign targeting both enzymes. A unique aspect of our approach is the screening of both enzymes simultaneously using crystals of the bifunctional PRODH-GSALDH enzyme, proline utilization A (PutA). A 288-fragment library from Zenobia was screened *in crystallo* in cocktails of six fragments. Validation X-ray crystallography with individual fragments identified seven crystal hits distributed in the PRODH active site, GSALDH aldehyde substrate-binding site, and GSALDH NAD^+^ adenine-binding site. The fragment bound in the PRODH active site, 4-methoxybenzyl alcohol, is structurally distinct from all known PRODH inhibitors as it lacks an anionic anchor and stabilizes open conformations of the active site, motivating the study of eighteen analogs. In total, thirteen crystal structures with resolutions ranging from 1.32 Å to 1.80 Å were determined, resolving the poses and interactions of seven fragments from the Zenobia library and five analogs of 4-methoxybenzyl alcohol. These results expand the chemical space of probes targeting proline catabolic enzymes and provide new structural information for further inhibitor development.

## 1. Introduction

**Figure 1:**
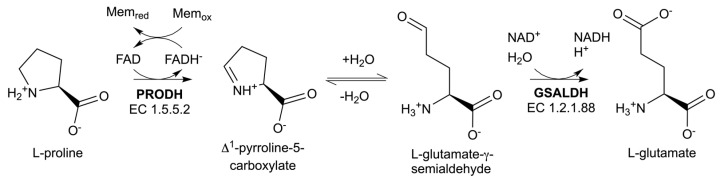
Reactions of proline catabolism.

Proline catabolism comprises the reactions catalyzed by proline dehydrogenase (PRODH) and L-glutamate-γ-semialdehyde (GSAL) dehydrogenase (GSALDH) [1]. PRODH is a flavoenzyme that catalyzes the FAD-dependent oxidation of L-proline to Δ^1^-pyrroline-5-carboxylate (P5C) (Figure 1). The hydrolysis of P5C generates GSAL, which is the substrate for GSALDH. Also known as ALDH4A1, GSALDH is a member of the aldehyde dehydrogenase (ALDH) superfamily and catalyzes the NAD^+^-dependent oxidation of GSAL to L-glutamate.

The arrangement of proline catabolic genes varies among organisms. In eukaryotes, the genes are distinct and encode separate PRODH and GSALDH enzymes. Both enzymes are localized to the mitochondria, with PRODH located in the inner mitochondrial membrane and GSALDH in the matrix. In contrast, in some bacteria, most notably Gram-negative bacteria, the *PRODH* and *GSALDH* genes are combined into the *putA* gene, which encodes a bifunctional enzyme, proline utilization A (PutA) [1].

PRODH is a target of inhibitor discovery because of its role in the metabolism of certain cancer cells. The role of PRODH in cancer is complicated and depends on cancer type and tumor microenvironment [2,3,4,5,6]. As a p53-induced anti-cancer protein, PRODH induces apoptosis and senescence through ROS signaling in different types of cancers, whereas PRODH promotes malignant phenotypes of certain tumors under stresses such as hypoxia. Breast cancer is an example of the latter. PRODH expression is upregulated in human metastatic breast tissue compared to primary breast tumor tissue [7]. Relevant to inhibitor discovery, the noncovalent PRODH inhibitor S-(-)-tetrahydro-2-furoic acid (THFA) was shown to inhibit the spheroidal growth of breast cancer cells and impair the formation of lung metastases in mouse models of breast cancer [7]. Also, mechanism-based inactivators that covalently modify the FAD of PRODH show activity in cancer cells [8,9].

The inhibition of GSALDH is also of interest. ALDHs are markers of cancer stem cells and may also play functional roles in the self-protection, differentiation, and/or expansion of stem cell populations [10]. Although ALDH1A1 and ALDH3A1 are most often associated with cancer stem cells [11,12], high ALDH4A1 expression has been reported in prostate cancer cells and primary cultures of human prostate cancer [13]. Inhibitors of ALDH4A1 could also potentially be used as pharmacological chaperones [14,15] to stabilize the misfolded variants of ALDH4A1 produced in patients with hyperprolinemia II, a metabolic disorder caused by mutations in the *ALDH4A1* gene which can result in neurological problems, including intellectual disability [16,17].

**Figure 2:**
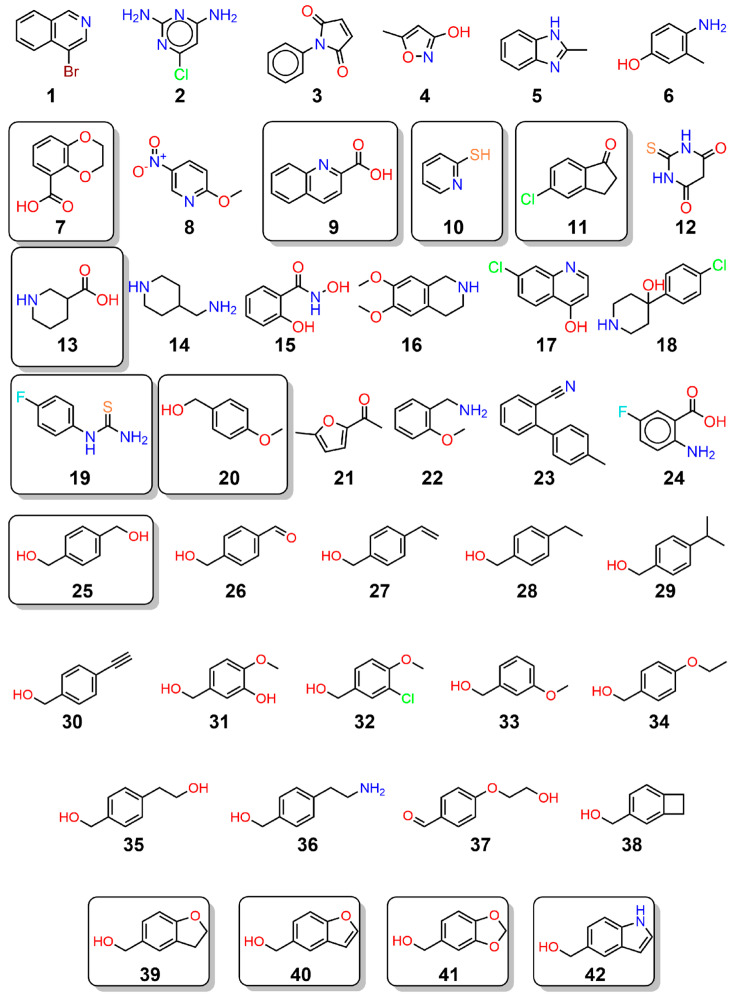
Compounds used in the validation and follow-up X-ray crystallography experiments. The boxes indicate compounds for which a strong electron density was observed and crystal structures were determined and deposited in the PDB.

Herein we report the results of a crystallographic fragment-screening campaign of proline catabolic enzymes. A unique aspect of our approach was the use of the bifunctional enzyme PutA as the target, which enabled the simultaneous screening of both PRODH and GSALDH. Crystals of PutA from *Sinorhizobium meliloti* (SmPutA, UniProt F7X6I3) were soaked with the Zenobia Express-Zen-Core 288™ library in cocktails of six fragments. Validation X-ray crystallography using twenty-four individual compounds (**1**–**24** in Figure 2) revealed seven crystal hits distributed in the PRODH active site, GSAL-binding site, GSALDH NAD^+^-binding site, and surface sites (**7**, **9**, **10**, **11**, **13**, **19**, and **20**). The fragments in the GSAL site engage known elements of aldehyde substrate recognition and provide starting points for the development of competitive inhibitors of GSALDH. Fragment **20** in the PRODH active site is unique among PRODH inhibitors and motivated the study of eighteen analogs (**25**–**42**), which resulted in five additional crystal structures (**25**, **39**–**42**). The inhibition of enzyme activity by the crystal hits was also tested. These results expand the chemical space of probes targeting proline catabolic enzymes and provide new structural information for further inhibitor development.

## 2. Results

### 2.1. Crystallographic Fragment Screening and Validation

Crystallographic fragment screening was performed by soaking crystals of SmPutA with compounds from the Zenobia Express-Zen-Core 288™ X-ray Mix of Six Fragment Screen. Each crystal was soaked for 48 h in a cocktail of six fragments at 10 mM of each compound (60 mM total fragment concentration). The soaks were performed in duplicate. Sixty-one X-ray diffraction data sets were collected. Data sets were not obtained for two wells (B1, H2) due to the deterioration of the crystals during soaking. The high-resolution limits of the data sets ranged from 1.53 Å to 2.98 Å with a median of 1.75 Å and a mean of 1.80 ± 0.25 Å. Eight data sets had high-resolution limits better than 2 Å. Visual inspection of the electron density maps suggested hits from the following wells of the library: A1, B4, D4, E3, E4, and H1 (the fragment cocktail compositions are provided in a spreadsheet in Supplementary Materials). The clearest hits were compounds **7** and **13**, bound to the GSAL-binding site of the GSALDH active site. The other hits were characterized by ambiguous electron density features that required follow-up validation X-ray crystallography to identify the fragments and their poses.

The hits from cocktailed fragment screening were validated by soaking SmPutA crystals with individual compounds (**1–24**). These experiments resulted in the determination of eight crystal structures, resolving the poses and interactions of seven fragments, bound to the GSAL pocket of the GSALDH active site (**7**, **13**, **20**), the NAD^+^ adenine site in the GSALDH active site (**9**, **10**, **11**, **19**), the PRODH active site (**20**), and surface sites (**9**). The high-resolution limits of the structures, binding locations of the fragments, and PDB deposition IDs are listed in Table 1. The complete X-ray diffraction data processing and refinement statistics are listed in Table S1.

### 2.2. Poses and Interactions of Fragments **7**, **13**, and **20** Bound to the GSAL-Binding Site

**Figure 3:**
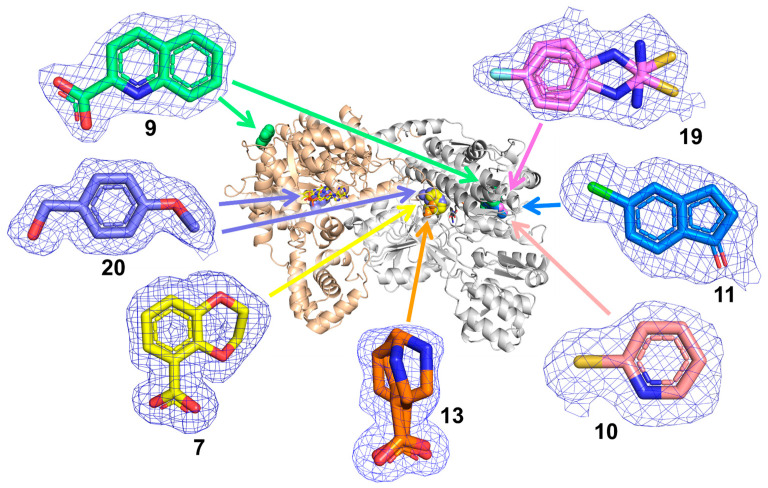
The locations of validated hit compounds in the tertiary structure of SmPutA. The mesh represents polder omit maps (4σ). The PRODH half of SmPutA is shown on the left in beige, and the GSALDH half is shown on the right in white. FAD is shown as yellow sticks. NAD is shown as gray sticks.

Fragments **7** and **13** were bound to the GSAL substrate-binding site of the GSALDH active site. The electron density for both fragments was unambiguous and supported the inclusion of the fragments at high occupancy (Figure 3). The refined occupancies of **7** were 0.83 and 0.86 in chains A and B of the asymmetric unit, respectively. Fragment **13** was modeled as a mixture of the *R* and *S* enantiomers, with occupancies of 0.55/0.45 (R/S) in chain A and 0.53/0.47 (R/S) in chain B.

**Figure 4:**
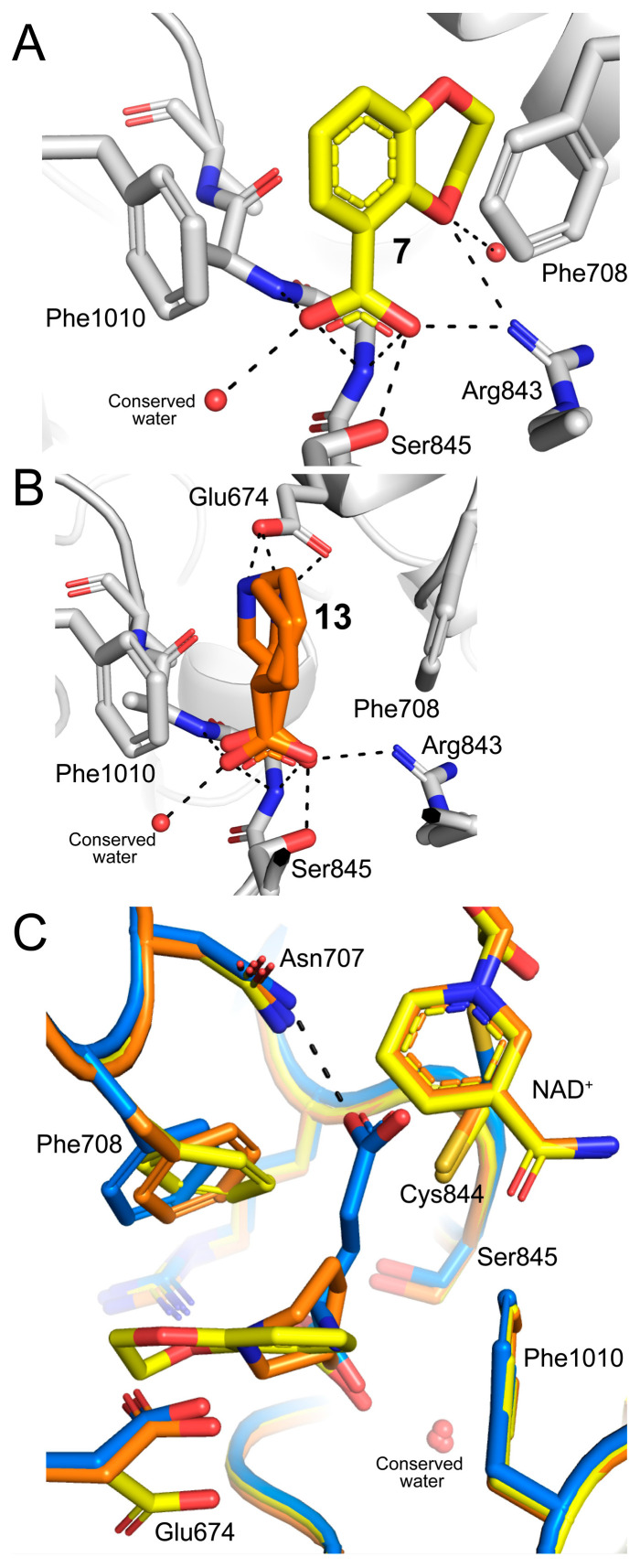
Poses and interactions of **7** and **13**: (**A**) fragment **7**; (**B**) fragment **13**; and (**C**) comparison of the binding modes of **7** (fragment, protein, and NAD^+^ in yellow, PDB ID 9DL2) and **13** (fragment, protein, and NAD^+^ in orange, PDB ID 9DL6) with the product L-glutamate (glutamate and protein in blue, PDB ID 9BBO).

Fragments **7** and **13** engaged well-known recognition elements of ALDH superfamily enzyme active sites (Figure 4A,B) [18,19]. The carboxylates formed hydrogen bonds with the backbone of the anchor loop (1000s loop in SmPutA). The carboxylates also formed an ion pair with Arg843 and a hydrogen bond with Ser845. The heterocycles were flanked by two Phe side chains, which formed the so-called aromatic box of ALDH active sites. All these recognition elements have also been observed in the structures of mouse GSALDH and SmPutA complexed with the product glutamate (PDB IDs 4V9K and 9BBO).

The fragments also exhibited interactions not found in the product complex. The dioxane ring of **7** forms a hydrogen bond with Arg843 (Figure 4A). The amine group of **13** donated a hydrogen bond to Glu674; both enantiomers showed this interaction (Figure 4B).

The fragments also differed from the substrate GSAL in that they did not occupy the oxyanion hole. In the glutamate complex, which is a model for the GSAL Michaelis complex, the side-chain carboxylate formed hydrogen bonds with a conserved asparagine (Asn707) and the backbone amino group of the catalytic cysteine (Cys844) (Figure 4C). These two interactions define the oxyanion hole of ALDH superfamily enzymes. The cyclic structures of **7** and **13** were perpendicular to the side chain of the glutamate product and, thus, could not access the oxyanion hole (Figure 4C).

**Figure 5:**
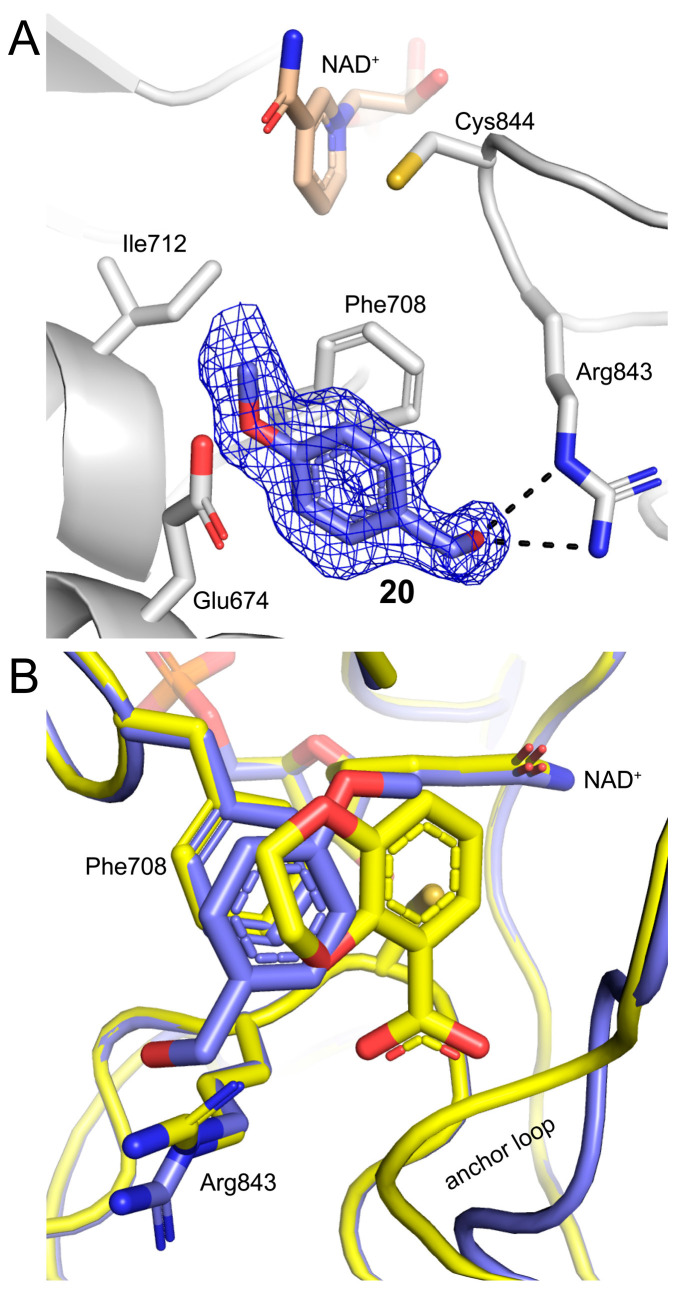
Fragment **20** in the GSALDH active site: (**A**) polder omit map (4σ) and interactions; and (**B**) comparison of the poses of **20** (fragment, protein, and NAD^+^ in blue, PDB ID 9DL9) and **7** (fragment, protein, and NAD^+^ yellow, PDB ID 9DL2).

One instance of fragment **20** was found in the GSALDH site. This feature was observed in only one chain of the two structures determined for this fragment (replicate structure 2 in Table 1 and Table S1). We note that the primary binding site for **20** is the PRODH site (*vide infra*). Nevertheless, the electron density for **20** in the GSALDH site was convincing and allowed modeling of the fragment at an occupancy of 0.71 in chain B (Figure 5A). Fragment **20** occupied the same space as the dioxane ring of **7** (Figure 5B). The hydroxyl of **20** forms a hydrogen bond to Arg843, while the phenyl ring was packed between one of the aromatic box residues (Phe708) and the aliphatic chain of Glu674 (Figure 5A). The O-methyl group contacts Ile712. Notably, **20** did not interact with the anchor loop and, thus, did not mimic the substrate GSAL, unlike **7** and **13**.

### 2.3. Poses and Interactions of Fragments **9**, **10**, **11**, and **19** Bound to the GSALDH NAD^+^-Binding Site

Fragments **9**, **10**, **11**, and **19** bound to the GSALDH NAD^+^-binding site and, more specifically, overlap with the adenine group of NAD^+^ (Table 1). Fragments **9** and **10** were modeled into this site with high occupancy in both chains of the asymmetric unit (*Q* = 0.85–1.00). Convincing electron density for **11** was observed only in chain A, and the fragment was modeled with an occupancy of 1.00. The density for **19** was strong in both chains but lacked detailed features to support a single-binding mode. Therefore, this fragment was modeled in two plausible conformations in each chain, with a total occupancy of 0.9 per chain.

**Figure 6:**
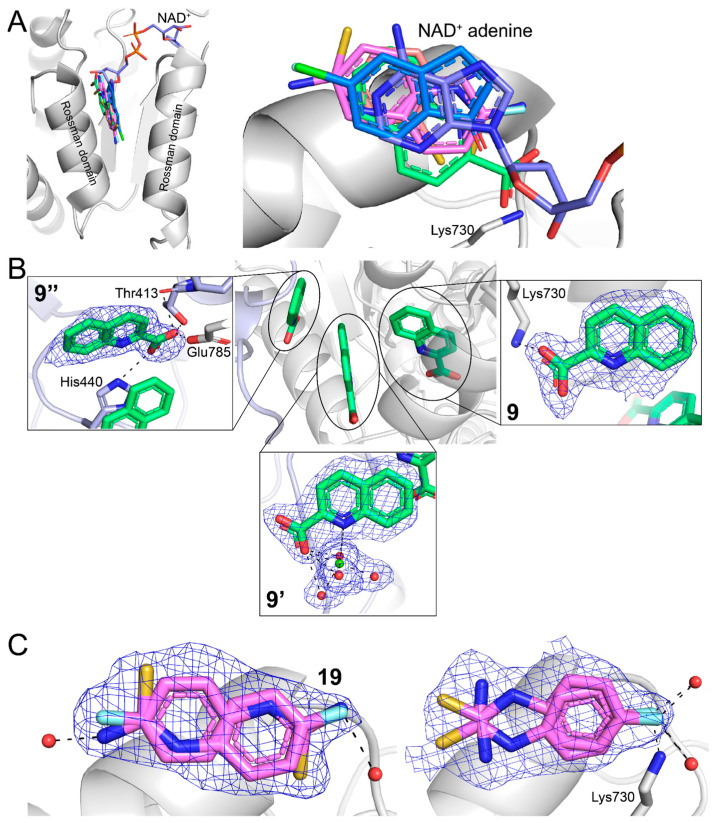
Fragments **9**, **10**, **11**, and **19** bound to the NAD^+^-binding site of the GSALDH active site: (**A**) overlap of all four fragments with the adenine of NAD^+^, where the NAD^+^ is from PDB entry 5KF6; (**B**) locations and polder omit maps (4σ) for fragment **9** bound to the adenine site (denoted by **9**) and two surface sites formed by crystal contacts (denoted **9’** and **9”**), where the protein in the asymmetric unit is white, and the symmetry-related chain is light blue; (**C**) the four conformations modeled for **19** in chain A (left) and chain B (right). The polder omit maps are shown (4σ).

Mimicking the adenine of NAD^+^, fragments **9**, **10**, **11**, and **19** were wedged between two α-helices of the Rossmann dinucleotide-binding domain (Figure 6A). One edge of the fragment made contact with β1 of the Rossman fold, while the other edge was exposed to solvent. The latter was analogous to the N6-N7 edge of adenine in NAD^+^.

The fragments in the adenine site formed relatively few electrostatic interactions with the enzyme. The carboxylate of **9** formed an ion pair with Lys730 (Figure 6B, right panel) and hydrogen bonds with water molecules. Fragments **10** and **11** formed hydrogen bonds only with water molecules.

As mentioned above, **19** was modeled in two conformations. In chain A, the two conformations differed by a rotation of 180° that swapped the thiourea and fluorine groups (Figure 6C, left). In chain B, the two conformations differed in the position of the thiourea groups (Figure 6C, right). In three of the four poses, the F atom formed hydrogen bonds with water molecules, while, in one of the poses, the F atom formed hydrogen bonds with Lys730.

### 2.4. Poses and Interactions of Fragment **9** Bound to a Surface Site

Electron density for two copies of **9** was observed on the surface of the protein in a crystal contact region where the NAD^+^-binding domain of one protein met the PRODH domain of another protein. We note that this interface is not present in the in-solution dimer, which was validated previously by small-angle X-ray scattering [20]. The two surface-bound fragments (**9’** (*Q* = 0.88) and **9”** (*Q* = 0.80) in Figure 6B) were packed between the instance of **9** in the adenine site and the PRODH domain of a symmetry-related molecule. One of the surface fragments participated in the octahedral coordination of a metal ion, which was modeled as Mg^2+^ since the crystallization solution contained 0.1 M MgCl_2_ (**9’** in Figure 6B). The coordination sphere of the Mg^2+^ ion consisted of O and N atoms of **9’** plus four water molecules. Although N is less common than O as a Mg^2+^ ligand [21], searching the PDB with the MESPEUS server reveals many high-resolution examples of histidine side chains coordinating Mg^2+^ (e.g., 3AYX) [22]. The numerous crystal contacts stabilizing **9’** and **9”** suggest that these sites are most likely artifacts of crystallization.

### 2.5. Poses and Interactions of Fragment **20** Bound to the PRODH Active Site

**Figure 7:**
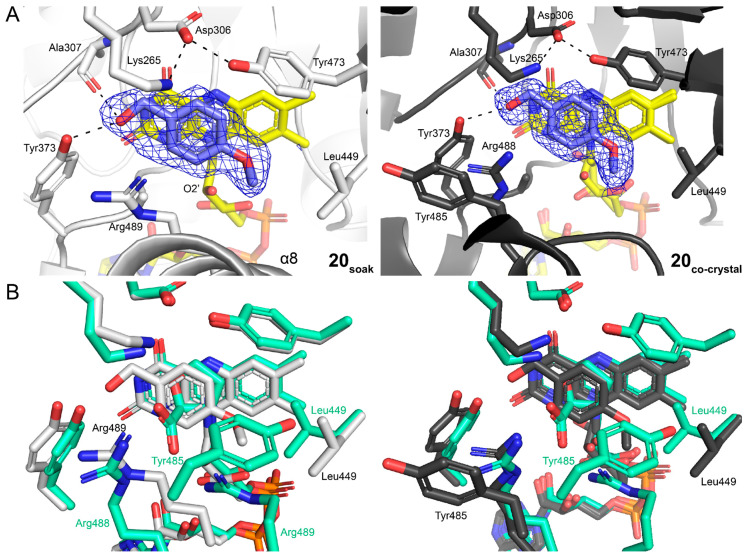
Fragment **20** bound to the PRODH active site: (**A**) electron density (polder omit, 4σ) and interactions for **20**, with replicate structures 1 and 2 shown in the left and right panels, respectively, and the FAD shown in yellow; and (**B**) comparison of the PRODH active sites complexed with **20** and the proline analog THFA (shown in green). Replicate structures 1 (white) and 2 (black) are shown in the left and right panels, respectively.

Fragment **20** was bound to the PRODH active site. Interestingly, the electron density for **20** in this location was not evident in the maps obtained from soaking crystals with fragment cocktails. Instead, diffuse electron density features presumably representing multiple compounds from well H1 were observed in the GSALDH active site. However, upon soaking crystals with **20** alone at the higher concentration of 67 mM, a clear electron density feature was observed in the PRODH active site (Figure 7A).

Two replicate structures of SmPutA complexed with **20** were determined. Replicate structure 1 (1.64 Å resolution) was obtained by soaking a crystal with 67 mM of **20** for 24 h. Upon observing a new PRODH active site conformation, we also co-crystallized SmPutA with 20 mM **20** and then included 67 mM **20** during cryoprotection (replicate structure 2, 1.32 Å resolution); the exposure of the crystal to 67 mM **20** during cryoprotection was brief (a few minutes). The fragment was modeled with occupancies of 0.66 and 0.78 in replicate structure 1 and 0.68 and 0.76 in replicate structure 2.

The poses of fragment **20** in the two structures were similar. Fragment **20** was bound to the L-proline substrate-binding site of the PRODH active site (Figure 7A). One face of the benzene ring stacked parallel against the *si* face of the FAD isoalloxazine. The other face of **20** made contact with the side chains of conserved lysine and arginine residues but was mostly unobstructed and exposed to a solvent-filled cavity that led to the GSALDH active site. The hydroxyl of **20** occupied the binding site of a conserved water molecule observed in PRODH structures. Apparently, the binding of **20** displaced the conserved water molecule. The hydroxyl of **20** formed hydrogen bonds to the phenol of Tyr373 and the carbonyl of Ala307 (Figure 7A). The methyl group of **20** occupied a hydrophobic pocket near a conserved leucine (Leu449). One edge of **20** made contact with the roof of the active site formed by Asp306 and Tyr473, while the other edge made contact with the O2’ hydroxyl of the FAD ribityl chain.

The FAD of PRODH is known to adopt different conformations in response to ligand binding and reduction [23]. The dominant FAD conformation in the presence of **20** appears to be the one associated with the binding of proline analog inhibitors and, by inference, the substrate L-proline. In this conformation (Michaelis complex conformation), the isoalloxazine, which was oxidized, was planar, and the O2’ hydroxyl of the ribityl chain pointed to the proline-binding site (Figure 7A). The O3’ and O4’ hydroxyls were located below the dimethylbenzene and pyrimidine parts of isoalloxazine, respectively. This conformation was observed at full crystallographic occupancy in replicate structure 1. Two conformations were observed in replicate structure 2. The Michaelis complex conformation was modeled with an occupancy of 0.59/0.72 (chains A/B), while the conformation associated with the unoccupied proline site was modeled at an occupancy of 0.41/0.28. This second FAD conformation differed by a crankshaft rotation of the ribityl chain that moved the O2’ hydroxyl below the pyrimidine ring of the isoalloxazine (Figure 7A, right-hand panel).

Although **20** occupied the proline site and stabilized the Michaelis complex FAD conformation, it did not fully mimic the substrate L-proline. Figure 7B compares the structures of SmPutA complexed with **20** and the proline analog THFA. Major differences between the two structures includes the side-chain rotamer of Leu449 and helix α8. In the THFA complex, Leu449 adopted rotamer #1, which allowed the close packing of the side chain against the aliphatic portion of the tetrahydrofuran ring of THFA. In contrast, in the complex with **20**, Leu449 flipped to rotamer #2 to avoid a steric clash with the O-methyl group of the fragment.

The differences in α8 conformations were substantial. This helix is a key structural element of the active site. In the THFA complex, as well as in other proline analog complexes, α8 was highly ordered and contributed three conserved side chains that made contact with the proline analog: Arg488 and Arg489 ion pair with the carboxylate of THFA, while Tyr485 was packed against the aliphatic portion of the tetrahydrofuran ring (Figure 7B). These interactions closed the active site and buried the proline analog. In contrast, the electron density for α8 in the complexes with **20** was weak, implying conformational flexibility. As a result, several side chains and entire residues of α8 were omitted from the final models. Thus, the active site when **20** was bound appeared to be more flexible and open compared to the THFA complex. Thus, the fragment screen revealed a new PRODH active site conformation.

### 2.6. Inhibition of GSALDH Activity by Hits from Crystallographic Screening

**Figure 8:**
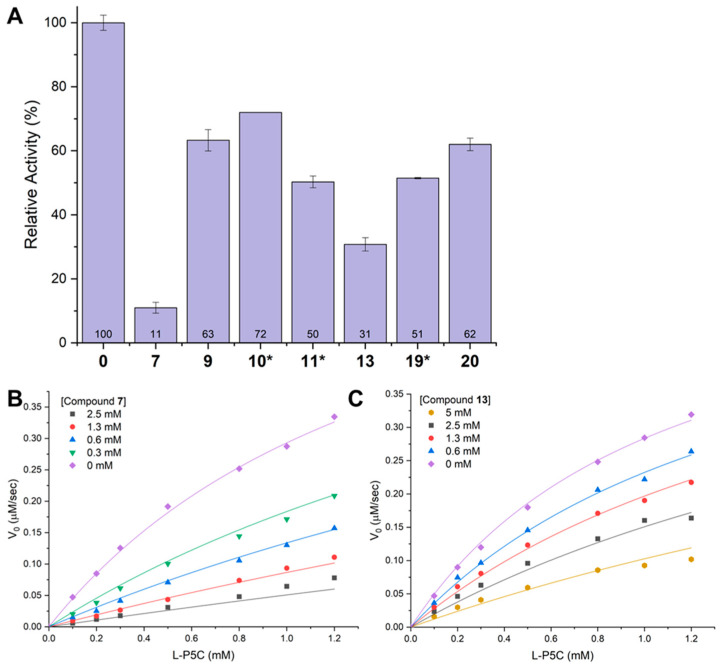
Inhibition of GSALDH activity of SmPutA by fragments: (**A**) Relative activity measurements. The bars represent the initial rate with L-P5C at 200 µM, NAD^+^ at 50 µM, and the fragment at 5 mM. The error bars represent the standard deviation from three technical replicates. The data are normalized to the rate of SmPutA in the absence of an inhibitor. * Due to low compound solubility, **11** was screened at 1 mM and **19** was screened at 3.8 mM; due to the absorbance of **10** at 340 nm, this fragment was tested at 0.25 mM. (**B**,**C**) Inhibition of GSALDH activity of SmPutA by **7** and **13** with L-P5C as the variable substrate and NAD^+^ fixed at 0.2 mM. The data were fitted to a global competitive inhibition model. The *K*_i_ for **7** is 0.32 mM ± 0.02 mM. The *K*_i_ for **13** is 1.5 mM ± 0.1 mM.

The seven hits from the crystallographic screening of the Zenobia library were tested for the inhibition of GSALDH activity. The fragments were included in the assay at 0.25–5 mM, depending on compound solubility or spectral interference at 340 nm. The most notable inhibition occurred with **7** and **13**, whose poses and interactions mimicked the substrate GSAL (Figure 8A). Then, **7** and **13** were further tested in assays performed with varying P5C/GSAL and fragment concentrations at a fixed NAD^+^ concentration of 0.2 mM. The data were fitted to a global competitive inhibition model, resulting in *K*_i_ values of 0.32 mM ± 0.02 mM for **7** and 1.5 mM ± 0.1 mM for **13** (Figure 8B,C).

### 2.7. Crystal Structures of SmPutA Complexed with Analogs of **20**

**Figure 9:**
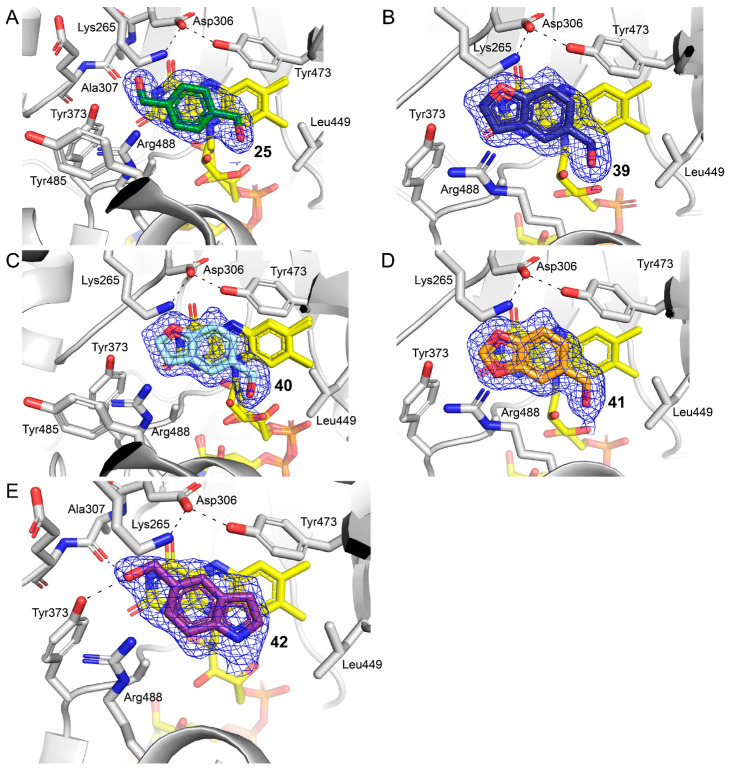
Electron density (polder omit, 4σ) and interactions for analogs of **20** bound to the PRODH active site: (**A**) fragment **25**; (**B**) fragment **39**; (**C**) fragment **40**; (**D**) fragment **41**; and (**E**) fragment **42**. The FAD is colored in yellow.

Compound **20** is chemically different from other PRODH inhibitors, which are proline analogs with an anionic anchor, and the structure of SmPutA complexed with **20** revealed a new, open PRODH active site conformation. We therefore explored this new class of PRODH binder by studying analogs of **20**. Eighteen analogs were used in the crystallization experiments (**25**–**42** in Figure 2). Five structures were determined at high-resolution limits of 1.32–1.47 Å, resolving the poses of compounds **25**, **39**, **40**, **41**, and **42** in the PRODH active site (see Table S1 for the crystallographic statistics). The electron density for these compounds was strong and covered the entire ligand (Figure 9). For all but compound **25**, the ligand was modeled in both chains in the asymmetric unit; compound **25** was modeled only in chain B. The occupancies of **39**–**42** ranged from 0.67 to 0.88; the occupancy of **25** was 0.68.

Compound **25** was isostructural to **20**, except **25** had two alcohol groups, whereas **20** had one alcohol group and a methoxy group. Interestingly, this minor difference affected the interactions of the fragment with the enzyme. Indeed, **25** was bound to the same location as **20**, stacking in parallel against the *si* face of the FAD isoalloxazine (Figure 9A). As with replicate structure 2 of fragment **20**, the electron density for the FAD was consistent with two conformations. One of the alcohol groups of **25** bound to the conserved water site, while the other was in the nonpolar pocket. The conformation of the former alcohol group was different from that of **20**. While the hydroxyl of **20** was positioned optimally to form hydrogen bonds with Ala307 and Tyr373, the hydroxyl of **25** was rotated by 180° so that it would form hydrogen bonds only with Ala307. In this conformation, the hydroxyl could not form a hydrogen bond with Tyr373, and, furthermore, the adjacent methylene group clashed with the hydroxyl of Tyr373 (C–O distance of 3.0 Å). The other hydroxyl of **25** (the one in the nonpolar pocket) did not form a hydrogen bond with the enzyme. Thus, the replacement of the methoxy of **20** with an alcohol group, paradoxically, diminished hydrogen bonding with the enzyme.

Compounds **39**–**42** were structurally similar bicyclic 5-6 systems consisting of benzyl alcohol fused to a five-membered ring heterocycle containing either O (**39**–**41**) or N (**42**). As with **20** and **25**, the ring system of these compounds stacked against the *si* face of the FAD isoalloxazine. Unexpectedly, the hydroxyls of **39**-**41** did not occupy the conserved water site and instead pointed to the nonpolar pocket near Leu449 (Figure 9B–D). The hydroxyl groups of these compounds did not form hydrogen bonds to the enzyme, similar to **25**.

The pose of **42** was distinctly different from the other bicyclic 5-6 compounds. Compound **42** was flipped by 180° so its hydroxyl group occupied the conserved water pocket, and the five-membered heterocycle occupied the nonpolar pocket (Figure 9E). The hydroxyl of **42** formed hydrogen bonds with both the carbonyl of Ala307 and the phenol of Tyr373. Thus, the alcohol group of this compound was optimally oriented for hydrogen bonding, similar to **20**. We validated the modeling of **42** by refining four alternative “flipped” poses in which the hydroxyl group was in the nonpolar pocket, like **39**–**41** (Figure S1). The flipped poses had a lower correlation coefficient with the polder map, calculated as CC(1,3), indicating a worse fit to the map [24]. For example, CC(1,3) degrades from 0.92 for the pose in Figure 9E to 0.82–0.86 for the flipped poses. Also, the refined occupancies of the fragment decreased in three of the four alternative refinements (Figure S1). Furthermore, flipping the fragment resulted in strong positive and negative *F*_o_-*F*_c_ features on the ligand (Figure S1). These results validated the pose of **42** shown in Figure 9E.

### 2.8. Inhibition of PRODH Activity by **20** and Its Analogs

**Figure 10:**
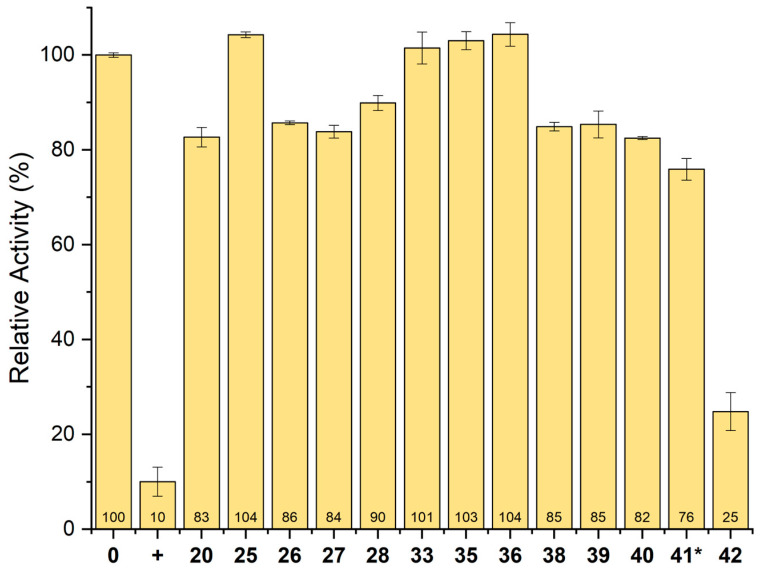
Inhibition of PRODH activity by **20** and its analogs. The bars represent the initial rate with L-proline at 25 mM, menadione at 0.1 mM, *o*-AB at 4 mM, and the fragment at 5 mM. The error bars represent the standard deviation from three technical replicates. The data are normalized to the rate of SmPutA in the absence of an inhibitor. **+** represents activity in the presence of the positive control, THFA (5 mM). * Due to low compound solubility, **41** was screened at 3 mM. Also, because of aqueous solubility limitations, the assays for **38** and **42** included 0.5% DMSO.

Fragment **20** and twelve analogs were tested for the inhibition of PRODH activity. The analogs tested included the five strong crystal hits plus seven others for which a weak electron density suggested the possibility of binding but was insufficient for structure determination. An assay with the known inhibitor THFA was included as a positive control. Each compound was included in the assay at 5 mM, except for **41**, which was used at 3 mM due to solubility issues. THFA lowered PRODH activity to 10% relative to having no inhibitor (Figure 10). Fragment **42** produced a notable lowering in activity to 25%.

## 3. Discussion

Here, we described a crystallographic fragment-screening campaign of the proline catabolic enzymes PRODH and GSALDH. A unique aspect of this study was the simultaneous screening of two enzymes in a single crystal, which was possible using the bifunctional PRODH-GSALDH enzyme, PutA. We identified binders of both PRODH and GSALDH. All the meaningful crystallographic hits targeted an active site, either the proline-binding site of PRODH, the NAD^+^ adenine site of GSALDH, or the GSAL substrate site of GSALDH (Table 1). One fragment (**9**) was bound to a remote site; however, this association with the protein appeared to have been enabled by crystal packing interactions.

The two fragments that bound to the GSAL site (**7**, **13**) were notable in that they inhibited enzyme activity. We note that it is common in crystallographic fragment screening to find compounds that bind to the active site and yet do not inhibit enzyme activity, presumably because the affinity is below the detection limit of the enzyme activity assay [25,26]. The competitive inhibition constants of 0.32 mM for **7** and 1.5 mM for **13** were better than those of proline and hydroxyproline stereoisomers, which were in the range of 1.3–30 mM [27]. Possible strategies for developing new GSALDH inhibitors include testing analogs of **7** and **13** and template docking, a virtual ligand screening approach which leverages high-resolution crystal structures of target–fragment complexes to reveal key interactions in the active site which are used to seed docking [28].

Our work provides a proof of concept for using crystallographic fragment screening to develop inhibitors of ALDH superfamily enzymes. The human ALDH superfamily comprises 19 enzymes, which catalyze the NAD^+^-dependent oxidation of numerous substrates, including small aldehydes such as acetaldehyde, amino acid derivatives, and lipids [29,30,31]. ALDHs play important roles in the detoxification of reactive aldehydes, amino acid metabolism, embryogenesis and development, neurotransmission, oxidative stress, and cancer. Some ALDH isoforms are being studied as potential cancer therapy targets [11,32]. Also, mutations in certain *ALDH* genes are associated with inherited metabolic diseases. Based on our experience here with GSALDH, fragment screening could be a productive strategy for developing new inhibitors and pharmacological chaperones for other ALDHs.

The discovery of fragment **20** as a PRODH binder is potentially significant in chemical probe development targeting this enzyme. All previously known PRODH inhibitors feature an anionic anchor, which interacts with conserved arginine and lysine residues. Most of these inhibitors have a carboxylate anchor and are close proline analogs, with the best example being THFA [33]. We recently showed that the sulfonate group also serves as an anchor, as demonstrated by the structure of PRODH complexed with 1-hydroxyethane-1-sulfonate [34]. Inhibitors with anionic anchors stabilize a tightly closed conformation of the active site in which the inhibitor is surrounded by several side chains and the FAD isoalloxazine. The congested nature of the closed active site restricts the size of compounds that can be accommodated, and the elaboration of proline analogs as an inhibitor discovery strategy has been challenging. Fragment **20** and its analogs (**25**, **39**–**42**) are notable because they lack an anionic anchor and are bound to an open form of the active site. The latter feature suggests that it may be possible to identify larger analogs of these compounds with improved affinity.

Interest in **20** as a novel PRODH binder motivated the study of several analogs. Indeed, **20** has limited interactions with the enzyme, which is expected for a fragment [35,36]. Although limited in number, the interactions formed by fragments are thought to be of a “high quality” and provide a basis for fragment-to-lead optimization. The high quality interactions of **20** appear to be the hydrogen bonds with a backbone carbonyl and tyrosine side chain. These interactions are possible because the hydroxyl occupies the conserved water site. Additionally, displacement of the conserved water molecule may be considered another high-quality interaction, which is entropic in origin. Fragment **42** also forms these hydrogen bonds. Surprisingly, **25**, **39**, **40**, and **41** do not form these bonds, owing to a 180° rotation of the alcohol group of **25** and flipping of the other compounds so that the hydroxyl occupies the nonpolar pocket instead of the conserved water site. The notable inhibition of PRODH activity by **42** supports the notion that the aforementioned hydrogen bonds are important for binding. Testing analogs of **42** and template docking based on the SmPutA-**42** structure could be promising inhibitor design strategies.

Finally, our experience with fragment **20** demonstrates a potential pitfall of cocktailing fragments. We used a screen in which crystals were soaked with solutions containing six fragments, with each fragment present at 10 mM. In the initial cocktail soak, the electron density for **20** in the PRODH site was not evident. Instead, a diffuse electron density was observed in the GSALDH active site. Surprisingly, during follow-up crystal-soaking studies using compound **20** alone at 67 mM, we observed strong and reproducible electron density for the fragment in the PRODH active site, which led to a novel PRODH inhibitor, compound **42**. These results support the assertion of Schiebel et al. that the use of fragment cocktails can lead to artifacts such as reduced occupancy, crystal damage, and interfragment reactions, which diminish the success rate of fragment screening [25].

## 4. Materials and Methods

### 4.1. Protein Production

The bifunctional PRODH-GSALDH enzyme, proline utilization A (PutA), from *Sinorhizobium meliloti* (SmPutA, UniProt F7X6I3) was used in the X-ray crystallography and enzyme activity assays. SmPutA was expressed in *Escherichia coli* BL21(DE3) cells and purified using immobilized metal affinity chromatography, followed by anion exchange chromatography and size-exclusion chromatography, using procedures similar to those described previously [20,23,27,37]. The N-terminal His tag was removed with TEVP after the affinity chromatography step. The buffer used in size-exclusion chromatography contained 50 mM Tris, 150 mM NaCl, 0.5 mM EDTA, and 0.5 mM TCEP at pH 7.5. Fractions from size-exclusion chromatography were concentrated to 12 mg/mL, distributed into 50 µL aliquots, flash-frozen in liquid nitrogen, and stored at −80°C.

### 4.2. Crystallization, Crystal Soaking, and Co-Crystallization

Fragment screening was performed by soaking crystals of SmPutA. The crystals were grown using procedures similar to those described previously [20,23,27,37]. Briefly, SmPutA (6 mg/mL) was incubated with 5 mM NAD^+^ and used in sitting drop vapor diffusion crystallography at 13 °C. The reservoir solution contained 0.1–0.4 M ammonium sulfate, 0.1 M sodium formate, 0.1 M MgCl_2_, 0.1 M HEPES at pH 7.5, and 16–26% (*w*/*v*) PEG 3350. Crystal drops were streaked with SmPutA microcrystals. A minimum of 96 crystals were needed to perform the entire fragment screen in duplicate.

The Zenobia Express-Zen-Core 288™ X-ray Mix of Six Fragment Screen (Zenobia Fragments, a division of Zenobia Therapeutics, San Diego, CA, USA) was purchased in a 48-well tray format. Each well contained the dried-down residue of six fragments. The names and SMILES of the compounds are provided in a spreadsheet in Supplementary Materials. The fragments were resuspended by adding 10 µL of crystal stabilization buffer containing 0.2 M ammonium sulfate, 0.1 M sodium formate, 0.1 M MgCl_2_, 0.1 M HEPES at pH 7.5, and 20% (*w*/*v*) PEG 3350, so that the final fragment concentration was 10 mM per fragment (60 mM total of the six fragments). Two SmPutA crystals were transferred into each well and soaked for 48 h at 13 °C. The soaked crystals were flash-cooled in liquid nitrogen after the addition of 13% (*v*/*v*) PEG 200.

To validate the fragments contributing to ligand electron density features in the fragment screen, individual compounds were purchased and used in crystal soaking and co-crystallization. A total of 24 compounds were tested in these follow-up experiments (compounds **1**–**24** in Figure 2). In addition, eighteen analogs of **20** were also studied (**25**–**42** in Figure 2). The compound concentrations used in soaking and co-crystallization are listed in Table S2. Except for **11** and **42**, the compounds were dissolved in 1 M Tris pH 8.5. Compound **11** was dissolved in 100% DMSO and then diluted into the cryo-buffer for crystal soaking. Compound **42** was dissolved in 50% (*w*/*v*) PEG 3350 at 200 mM for the crystallization studies to overcome the lack of solubility in Tris buffer.

### 4.3. X-Ray Crystal Structure Determination

Shutterless X-ray diffraction data were collected at the NECAT beamlines 24-ID-C and 24-ID E of the Advanced Photon Source and beamlines 4.2.2 and 8.2.1 of the Advanced Light Source. The data were processed with automated processing pipelines, which use XDS for indexing, integration, and scaling [38], POINTLESS for space group determination [39], AIMLESS for merging [40], and CTRUNCATE for the conversion of intensities to amplitudes and the generation of a test set of reflections (2% or 5%) for *R*_free_ calculations [41]. The space group is *P*2_1_ with a dimer in the asymmetric unit.

The initial phases were calculated by Fourier synthesis in Phenix [42] using a model derived from PDB entry 5KF6 or 6X9A. Phenix was used for crystallographic refinement. The B-factor model consisted of one TLS group per protein chain and isotropic B-factors for non-hydrogen atoms. Coot was used for iterative model building [43]. Electron density features representing fragments were identified by manual inspection of the 2*F*_o_-*F*_c_ and *F*_o_-*F*_c_ maps in both active sites, as well as by analysis with the blob picker and difference map peak tools in Coot. The data processing and refinement statistics are listed in Table S1.

### 4.4. Enzyme Inhibition Assays

Enzyme activity assays were performed in 96-well plates in a BioTek Epoch 2 microplate spectrophotometer (Agilent, Santa Clara, CA, USA) at 23 °C. The total assay volume was 200 µL. The PRODH activity assay measured the dihydroquinazolinium compound formed by the reaction of the product P5C with ortho-aminobenzaldehyde (*o*-AB) by absorbance at 443 nm (ε_443_ = 2.59 mM^−1^cm^−1^). The electron acceptor menadione was included in the assay to allow for the catalytic cycling of PRODH by oxidizing the reduced FAD. The assays contained final concentrations of 4 mM *o*-AB and 0.1 mM menadione in a buffer of 20 mM MOPS pH 7.5 and 10 mM MgCl_2_. For the analysis of inhibition, 25 mM L-proline and the inhibitor were pipetted into the 96-well plate and then a master mix including enzyme, menadione, and *o*-AB in the assay buffer was added to the plate by a multichannel pipette to initiate the reaction. The final concentration of enzyme in the assay was 200 nM. The initial rates were estimated from a linear regression of the first 30 min of the progress curve using Origin 2020 version 9.7.0.188 software.

The GSALDH activity of SmPutA in the presence of inhibitors was measured by monitoring NADH production at 340 nm with L-P5C as the substrate. The assay buffer contained 100 mM sodium phosphate at pH 7 and 1 mM EDTA. The SmPutA concentration in each assay was 230 nM. The initial rates were estimated from the linear regression of the first 5 min of the progress curve. To estimate the inhibition constants, L-P5C was the variable substrate, and the initial rate as a function of both the variable substrate and the inhibitor concentrations was fitted globally to the standard model of competitive inhibition using Origin 2020 version 9.7.0.188 software.

## Figures and Tables

**Table 1:** Resolution of structures and binding locations of fragments.

Fragment	Resolution (Å)	Binding Location	PDB ID
**7**	1.55	GSAL site	9DL2
**9**	1.77	NAD^+^ adenine site, surface	9DL3
**10**	1.80	NAD^+^ adenine site	9DL4
**11**	1.77	NAD^+^ adenine site	9DL5
**13**	1.42	GSAL site	9DL6
**19**	1.72	NAD^+^ adenine site	9DL7
**20** (replicate 1)	1.64	PRODH active site	9DL8
**20** (replicate 2)	1.32	PRODH active site, GSAL site	9DL9
**25**	1.39	PRODH active site	9E0A
**39**	1.47	PRODH active site	9E0B
**40**	1.32	PRODH active site	9E0C
**41**	1.33	PRODH active site	9E0D
**42**	1.37	PRODH active site	9E0E

## Data Availability

The coordinates and structure factor amplitudes for the crystal structures were deposited in the Protein Data Bank with the following accession codes: **7**, 9DL2; **9**, 9DL3; **10**, 9DL4; **11**, 9DL5; **13**, 9DL6; **19**, 9DL7; **20** (replicate 1), 9DL8; **20** (replicate 2), 9DL9; **25**, 9E0A; **39**, 9E0B; **40**, 9E0C; **41**, 9E0D; and **42**, 9E0E.

## Supplementary Materials

The following supporting information can be downloaded at https://www.mdpi.com/article/10.3390/molecules29225408/s1, Table S1: X-ray diffraction data collection and refinement statistics; Table S2: Concentrations of inhibitors used in soaking, co-crystallization, and cryoprotection; Figure S1: Refinement of four flipped poses of **42**; and Spreadsheet with the names and SMILES of Zenobia fragments.
