# Supplementary material for: Crystallographic Fragment Screening of a Bifunctional Proline Catabolic Enzyme Reveals New Inhibitor Templates for Proline Dehydrogenase and L-Glutamate-γ-semialdehyde Dehydrogenase
Source: Molecules. 2024 Nov 16;29(22):5408. doi: 10.3390/molecules29225408 (PMC11596857; doi:10.3390/molecules29225408)
Supplement: Supplementary file 1 [file molecules-29-05408-s001.zip › molecules-3301004-supplementary.pdf]

SUPPLEMENTARY INFORMATION

Crystallographic Fragment Screening of a Bifunctional Proline Catabolic Enzyme Reveals New Inhibitor Templates for Proline Dehydrogenase and L-Glutamate-γ-semialdehyde Dehydrogenase

Kaylen R. Meeks [1], Alexandra N. Bogner [1,†], Jay C. Nix [2], John J. Tanner [1,3,*]

1 Department of Biochemistry, University of Missouri, Columbia, MO 65211, USA; kmcbp@missouri.edu (K.R.M.); bogneral@gmail.com (A.N.B.)
2 Molecular Biology Consortium, Advanced Light Source, Lawrence Berkeley National Laboratory, Berkeley, CA 94720, USA; jcnix@lbl.gov
3 Department of Chemistry, University of Missouri, Columbia, MO 65211, USA
* Correspondence: tannerjj@missouri.edu; Tel.: +1-573-884-1280
† Current address: Lilly Biotechnology Center, Eli Lilly and Company, San Diego, CA 92121, USA.

Table of Contents

**Table S1.** X-ray diffraction data collection and refinement statistics.	S2-S7
**Table S2.** Concentrations of fragments used in soaking, co-crystallization and cryoprotection.	S8
**Figure S1.** Refinement of four flipped poses of **42**.	S9

Table S1. X-ray diffraction data processing and refinement statistics

	**7**	**9**	**10**	**11**
Beamline	ALS 8.2.1	ALS 4.2.2	ALS 4.2.2	ALS 8.2.1
Date Collected	2023-10-21	2023-06-30	2023-07-01	2023-10-20
Space group	$P2_1$	$P2_1$	$P2_1$	$P2_1$
Unit cell parameters (Å,°)	a = 100.40<br>b = 101.72<br>c = 126.29<br>β = 106.23	a = 100.96<br>b = 102.33<br>c = 127.06<br>β = 105.96	a = 101.16<br>b = 102.37<br>c = 127.50<br>β = 106.29	a = 100.54<br>b = 101.08<br>c = 125.76<br>β = 106.31
Wavelength (Å)	1.000034	1.072156	1.000	1.000024
Resolution (Å)	48.20-1.55 (1.58-1.55)	48.53-1.77 (1.80-1.77)	48.55-1.80 (1.83-1.80)	48.25-1.77 (1.80-1.77)
Observations[a]	1192213 (61933)	754438 (20177)	1555199 (79769)	812644 (41392)
Unique reflections[a]	345973 (17375)	230404 (8678)	230029 (11331)	232824 (11546)
$R_{merge}(I)$[a]	0.046 (0.716)	0.079 (0.917)	0.074 (1.190)	0.058 (0.834)
$R_{meas}(I)$[a]	0.055 (0.844)	0.094 (1.192)	0.081 (1.286)	0.069 (0.980)
$R_{pim}(I)$[a]	0.029 (0.42)	0.051 (0.749)	0.031 (0.483)	0.037 (0.511)
Mean I/σ[a]	14.1 (1.6)	12.3 (0.8)	14.8 (1.6)	15.2 (1.6)
$CC_{1/2}$[a]	0.998 (0.650)	0.996 (0.426)	0.999 (0.628)	0.999 (0.577)
Completeness (%)[a]	98.3 (99.6)	95.5 (73.2)	99.8 (100.0)	99.3 (99.5)
Multiplicity[a]	3.4 (3.6)	3.3 (2.3)	6.8 (7.0)	3.5 (3.6)
No. of protein residues	2424	2397	2404	2394
No. of atoms				
Protein	18020	17786	17623	17513
FAD	212	212	106	106
NAD	88	-	-	-
Fragment	26	52	14	11
Water	2311	2214	1393	1772
R_{cryst}[a]	0.1674 (0.2706)	0.1767 (0.3020)	0.1940 (0.2844)	0.1731 (0.3046)
R_{free}[a]	0.1896 (0.2876)	0.2129 (0.3494)	0.2242 (0.3402)	0.2061 (0.3536)
R_{free} Test Set Size (%)	5	2	5	5

	7	9	10	11
RMSD bonds (Å)	0.006	0.006	0.006	0.006
RMSD angle (°)	0.867	0.844	0.823	0.821
Ramachandran plot[b]				
Favored (%)	98.17	97.94	98.07	97.94
Outliers (%)	0.04	0.13	0.04	0.00
Clashscore[b]	3.1 (98)	3.14 (98)	2.2 (99)	2.97 (99)
MolProbity Score[b]	1.10 (99)	1.12 (100)	1.03 (100)	1.10 (100)
Average B ($Å^2$)				
Protein	26.8	23.3	33.4	26.1
FAD	22.6	22.3	32.2	22.3
NAD	26.4	-	-	-
Fragment	27.7	28.7	36.7	35.6
Water	34.9	31.0	35.8	32.5
FAD Occupancy	1.00	1.00	1.00	1.00
NAD Occupancy	0.74, 0.75	-	-	-
Fragment Occupancy	0.83, 0.86	0.88, 0.88, 0.80, 0.85	1.00, 1.00	1.00
Coord. error[c]	0.17	0.23	0.25	0.21
PDB ID	9DL2	9DL3	9DL4	9DL5

[a]Values for the outer resolution shell of data are given in parenthesis. [b]From MolProbity. The percentile ranks for Clashscore and MolProbity score are given in parentheses. [c]Maximum likelihood-based coordinate error estimate from PHENIX.

Table S1-continued. X-ray diffraction data processing and refinement statistics

	**13**	**19**	**20-1**	**20-2**
Beamline	ALS 8.2.1	ALS 4.2.2	ALS 8.2.1	ALS 8.2.1
Date Collected	2024-04-25	2023-07-01	2023-10-20	2023-12-16
Space group	$P2_1$	$P2_1$	$P2_1$	$P2_1$
Unit cell parameters (Å,°)	a = 100.51<br>b = 101.83<br>c = 126.54<br>β = 106.43	a = 101.14<br>b = 101.87<br>c = 126.56<br>β = 106.38	a = 100.75<br>b = 101.30<br>c = 125.20<br>β = 106.34	a = 100.75<br>b = 101.96<br>c = 126.17<br>β = 106.32
Wavelength (Å)	1.00002	1.000018	1.000034	1.000090
Resolution (Å)	48.21-1.42<br>(1.44-1.42)	46.97-1.72<br>(1.75-1.72)	48.34-1.64<br>(1.67-1.64)	48.34-1.32<br>(1.34-1.32)
Observations[a]	2822743<br>(58042)	1679862 (89890)	1004747 (51748)	3574469 (80807)
Unique reflections[a]	419313 (12404)	259034 (12912)	291287 (14497)	538908 (17661)
$R_{merge}(I)$[a]	0.051 (1.538)	0.217 (2.356)	0.050 (0.757)	0.052 (1.720)
$R_{meas}(I)$[a]	0.056 (1.733)	0.238 (2.544)	0.059 (0.894)	0.056 (1.942)
$R_{pim}(I)$[a]	0.021 (0.785)	0.096 (0.953)	0.032 (0.470)	0.021 (0.886)
Mean I/σ[a]	18.1 (0.8)	6.2 (0.6)	15.1 (1.6)	16.6 (0.7)
$CC_{1/2}$[a]	0.999 (0.335)	0.981 (0.397)	0.999 (0.633)	0.999 (0.352)
Completeness (%)[a]	91.4 (54.9)	99.4 (99.8)	98.9 (99.4)	94.3 (62.5)
Multiplicity[a]	6.7 (4.7)	6.5 (7.0)	3.4 (3.6)	6.6 (4.6)
No. of protein residues	2434	2401	2407	2426
No. of atoms				
Protein	18269	17621	17795	18177
FAD	212	106	106	212
NAD	88	-	115	88
Fragment	36	44	20	30
Water	2422	1498	1804	2660
R_{cryst}[a]	0.1674 (0.3267)	0.2084 (3025)	0.1656 (0.2819)	0.1682 (0.3372)
R_{free}[a]	0.1889 (0.3211)	0.2400 (0.3131)	0.1925 (0.2937)	0.1878 (0.3677)
R_{free} Test Set Size (%)	5	2	5	5

	13	19	20-1	20-2
RMSD bonds (Å)	0.006	0.006	0.006	0.006
RMSD angle (°)	0.882	0.813	0.852	0.961
Ramachandran plot[b]				
Favored (%)	98.35	98.11	97.79	98.05
Outliers (%)	0.00	0.04	0.08	0.04
Clashscore[b]	3.38 (97)	2.38 (99)	3.04 (98)	3.2 (97)
MolProbity Score[b]	1.13 (98)	1.02 (100)	1.14 (99)	1.11 (98)
Average B ($Å^2$)				
Protein	25.9	30.5	26.4	23.8
FAD	20.0	28.4	21.4	18.7
NAD	22.1	-	29.2	19.1
Fragment	24.2	34.7	29.1	22.1
Water	33.8	34.5	34.1	33.0
FAD Occupancy	1.00	0.88-1.00	1.00	1.00
NAD Occupancy	0.97	-	0.78, 1.00	1.00
Fragment Occupancy	1.00, 1.00	0.90, 0.90	0.66, 0.78	0.68, 0.76, 0.71
Coord. error[c]	0.16	0.27	0.18	0.15
PDB ID	9DL6	9DL7	9DL8	9DL9

[a]Values for the outer resolution shell of data are given in parenthesis. [b]From MolProbity. The percentile ranks for Clashscore and MolProbity score are given in parentheses. [c]Maximum likelihood-based coordinate error estimate from PHENIX.

Table S1-continued. X-ray diffraction data processing and refinement statistics

	**25**	**39**	**40**	**41**	**42**
Beamline	ALS 8.2.1	ALS 8.2.1	ALS 8.2.1	ALS 8.2.1	ALS 8.2.1
Date Collected	2023-12-16	2023-12-16	2023-12-16	2023-12-16	2023-12-16
Space group	$P2_1$	$P2_1$	$P2_1$	$P2_1$	$P2_1$
Unit cell parameters (Å,°)	a = 100.71<br>b = 102.07<br>c = 126.18<br>β = 106.38	a = 100.53<br>b = 101.76<br>c = 126.02<br>β = 106.30	a = 100.37<br>b = 101.64<br>c = 125.92<br>β = 106.42	a = 100.29<br>b = 101.51<br>c = 125.58<br>β = 106.38	a = 100.46<br>b = 101.50<br>c = 125.33<br>β = 106.51
Wavelength (Å)	1.00008	1.00010	1.00009	1.00008	1.00008
Resolution (Å)	48.31-1.39 (1.41-1.39)	48.25-1.47 (1.50-1.47)	48.14-1.32 (1.34-1.32)	48.11-1.33 (1.35-1.33)	46.75-1.37 (1.39-1.37)
Observations[a]	3348544 (161024)	2812567 (143821)	3536195 (80388)	3608053 (107455)	3413325 (137607)
Unique reflections[a]	481320 (23190)	400330 (19700)	531732 (16856)	517023 (17707)	492578 (22701)
$R_{merge}(I)$[a]	0.074 (2.493)	0.098 (2.553)	0.060 (1.757)	0.065 (2.102)	0.077 (1.988)
$R_{meas}(I)$[a]	0.080 (2.694)	0.106 (2.746)	0.065 (1.975)	0.070 (2.299)	0.083 (2.175)
$R_{pim}(I)$[a]	0.030 (1.009)	0.040 (1.005)	0.025 (0.884)	0.026 (0.913)	0.031 (0.865)
Mean I/σ[a]	13.5 (0.7)	11.8 (0.8)	14.1 (0.7)	13.1 (0.6)	12.2 (0.7)
$CC_{1/2}$[a]	0.997 (0.332)	0.999 (0.370)	0.999 (0.369)	0.999 (0.353)	0.999 (0.285)
Completeness (%)[a]	98.3 (95.9)	97.2 (96.6)	93.9 (60.3)	93.9 (65.2)	97.8 (91.1)
Multiplicity[a]	7.0 (6.9)	7.0 (7.3)	6.7 (4.8)	7.0 (6.1)	6.9 (6.1)
No. of protein residues	2428	2423	2426	2421	2424
No. of atoms					
Protein	18117	17978	18096	17987	17917
FAD	212	106	212	106	106
NAD	88	88	88	88	88
Fragment	10	22	22	22	22
Water	2520	2181	2577	2387	2211
R_{cryst}[a]	0.1718 (0.3340)	0.1770 (0.3379)	0.1724 (0.3258)	0.1748 (0.3482)	0.1730 (0.3352)
R_{free}[a]	0.1909 (0.3399)	0.2000 (0.3541)	0.1904 (0.3263)	0.1930 (0.3415)	0.1920 (0.3368)
R_{free} Test Set Size (%)	5	5	5	5	5

	25	39	40	41	42
RMSD bonds (Å)	0.005	0.005	0.006	0.005	0.005
RMSD angle (°)	0.912	0.892	0.899	0.908	0.902
Ramachandran plot[b]					
Favored (%)	98.09	98.17	98.30	98.09	98.25
Outliers (%)	0.04	0.08	0.00	0.00	0.00
Clashscore[b]	2.57 (98)	3.01 (98)	2.92 (98)	3.1 (97)	2.67 (99)
MolProbity Score[b]	1.04 (99)	1.09 (99)	1.08 (99)	1.10 (98)	1.05 (99)
Average B (Å^2)					
Protein	24.7	24.8	23.1	24.8	23.9
FAD	19.9	20.7	18.3	20.9	20.1
NAD	19.4	19.9	18.7	19.7	18.7
Fragment	24.5	24.0	20.3	22.3	24.6
Water	33.4	32.2	31.7	32.4	31.8
FAD Occupancy	1.0	1.0	1.0	1.0	1.0
NAD Occupancy	1.0	1.0	1.0	1.0	1.0
Fragment Occupancy	0.68	0.79, 0.88	0.67, 0.76	0.68, 0.80	0.70, 0.77
Coord. error[c]	0.17	0.17	0.15	0.16	0.17
PDB ID	9E0A	9E0B	9E0C	9E0D	9E0E

[a]Values for the outer resolution shell of data are given in parenthesis. [b]From MolProbity. The percentile ranks for Clashscore and MolProbity score are given in parentheses. [c]Maximum likelihood-based coordinate error estimate from PHENIX.

Table S2. Concentrations of fragments used in co-crystallization and cryoprotection

Fragment	Co-crystallization (mM)	Cryoprotection (mM)
**7**	10	50
**9**	0	50
**10**	0	25
**11**	0	25
**13**	20	73
**19**	0	19
**20** (replicate structure 1)	0	67
**20** (replicate structure 2)	20	67
**25**	24	50
**39**	30	125
**40**	24	50
**41**	12	25
**42**	24	100

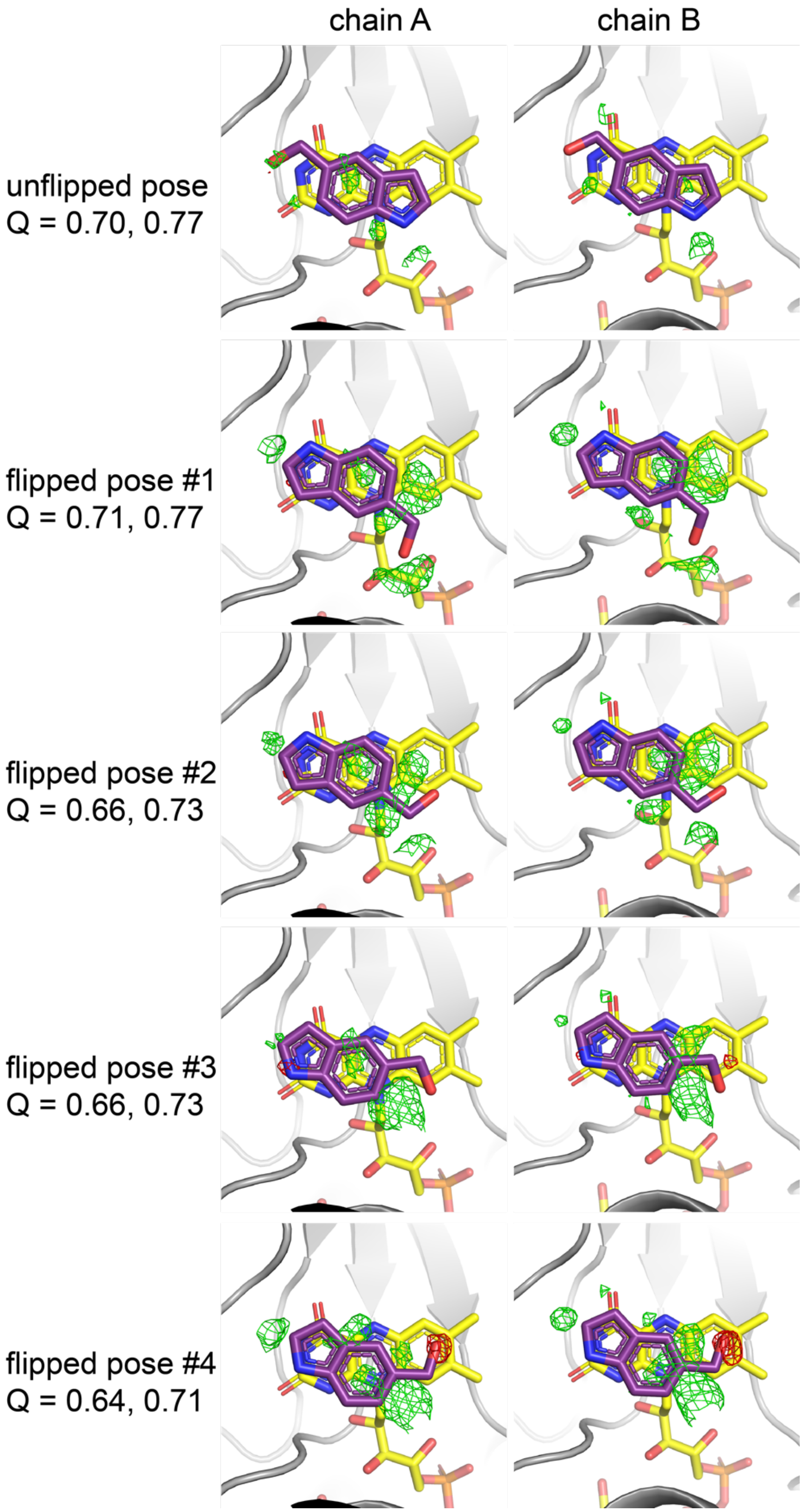

Figure S1. Refinement of four flipped poses of **42**. The green mesh represents the F_o-F_c map contoured at +3σ. The red mesh represents the F_o-F_c map contoured at -3σ. Refined occupancies for the ligand in chains A, B are listed.
